# Optimization (Central Composite Design) and Validation of HPLC Method for Investigation of Emtricitabine Loaded Poly(lactic-co-glycolic acid) Nanoparticles: *In Vitro* Drug Release and *In Vivo* Pharmacokinetic Studies

**DOI:** 10.1155/2014/583090

**Published:** 2014-01-30

**Authors:** Gurinder Singh, Roopa S. Pai

**Affiliations:** Department of Pharmaceutics, Faculty of Pharmacy, Al-Ameen College of Pharmacy, Near Lal-Bagh Main Gate, Hosur Road, Bangalore, Karnataka 560027, India

## Abstract

The objective of the current study is to develop nanoparticles (NPs) drug delivery system of emtricitabine solely using poly(lactic-co-glycolic acid) (PLGA) and evaluate its *in vitro *and *in vivo *release performance by systematically optimized HPLC method using Formulation by Design (FbD). NPs were evaluated for *in vitro *release and *in vivo *absorption study. The desired chromatographic separation was achieved on a Phenomenex C_18_ (250 mm × 4.6 mm I.D., 5 **μ**m) column, under isocratic conditions using UV detection at 280 nm. The optimized mobile phase consisted of a mixture of 40 mM phosphate dihydrogen phosphate buffer (pH 6.8), methanol, and 2% acetonitrile in a ratio of (83 : 15 : 2, v/v/v) at a flow rate of 1 mL/min. The linear regression analysis for the calibration curves showed a good linear correlation over the concentration range 0.040–2.0 **μ**g/mL, with retention time of 4.39 min. An average encapsulation efficiency of 74.34% was obtained for NPs. *In vitro *studies showed zero-order release and about 95% drug being released within 15 days in PBS (pH 7.4). In conclusion, the proposed optimized method was successfully applied for the determination of *in vitro *and *in vivo *release studies of emtricitabine NPs.

## 1. Introduction 

**Figure 1:**
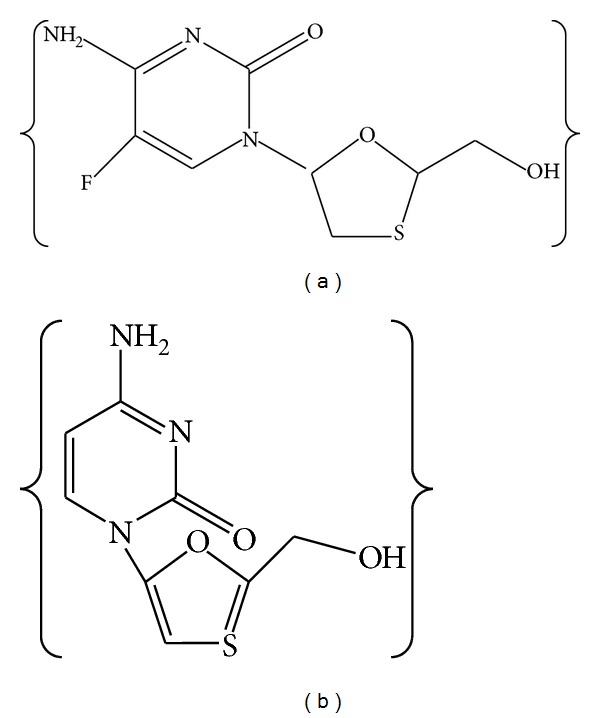
Chemical structure of emtricitabine (a) and lamivudine (b).

Emtricitabine (5-fluoro-1-(2R, 5S)-[2-(hydroxymethyl)-1,3-oxathiolan-5-yl]cytosine) (Figure 1(a)) is a potent deoxycytidine nucleoside reverse transcriptase inhibitor for the treatment of human immunodeficiency virus (HIV) infection [1, 2]. In adults, emtricitabine recommended therapeutic dose is 200 mg once a day (QD) [1]. Both *in vitro *[3] and *in vivo *[4] testing demonstrated that emtricitabine presents enough potential to be tested in the prevention of HIV-1, either alone or in combination [5–7].

Up to now, emtricitabine has been formulated as semisolid dosage form, microbicides (e.g., hydrophilic vaginal gels) [8], tablets [9, 10], capsules, and oral solutions [11]. In order to fully characterize emtricitabine formulations or delivery systems such as polymeric NPs, suitable and validated quantification methods are required to assess critical pharmaceutical characterization such as drug content, encapsulation efficiency, *in vitro* drug release, and *in vivo *absorption studies. A literature survey reveals several analytical methods are available for the determination of emtricitabine in bulk drug, plasma [12, 13] and pharmaceutical dosage forms, either alone or in combination with other antiretroviral drugs [14–18].

A few HPLC and a brief reference to one UPLC method for simultaneous determination of emtricitabine in combination with other antiretroviral drugs in human plasma have been described in the literature, mainly with the objective of method development for application to a bioequivalence study [19–21]. A simultaneous determination of emtricitabine and tenofovir in human plasma was described [22]. HPLC method was not suitable for detection of low emtricitabine concentration in human plasma [23]. HPLC-UV detection method was developed for simultaneous determination of emtricitabine and tenofovir in tablet dosage form with LOQ of above 0.091 *μ*g/mL [24]. A rapid RP-HPLC method for a combination of tenofovir disoproxil fumarate (TDF), emtricitabine (FTC), and efavirenz (EFV) was developed and subjected to forced degradation studies with LOQ of emtricitabine (FTC) being 1.19 *μ*g/mL [25]. A validated RP-HPLC method for the estimation of emtricitabine was not appropriate for detection of low emtricitabine concentration in capsules. In this method, the LOQ value was found to be 16.786 *μ*g/mL and retention time was more than 9.341 min [26].

A response surface methodology (RSM) approach was used to identify the optimum conditions for analysis during method development [27, 28]. Equation (1) represents a linear second-order model that describes a twisted plane with curvature, arising from the quadratic terms as follows:

(1) y=b0+b1x1+b2x2+b12x1x2+b11x12+b22x22,

Where *y*  is the experimental response to be optimized, *b*_0_ is a constant term and *b*_1_-*b*_5_ are coefficients of the linear terms, *x*_1_, *x*_2_ represent the main effect, *x*_1_^2^,   *x*_2_^2^ are the quadratic effect, and *x*_1_ *x*_2_ are the interaction effect. Data were analyzed by nonlinear estimation using Design Expert software 6.0.

In order to fully characterize the emtricitabine NPs formulation, a suitable and validated method is required for a critical assessment of pharmaceutical parameters such as drug content, encapsulation efficiency, and *in vitro* and *in vivo *release performance. Literature review reveals that HPLC methods have been reported for the quantitation of emtricitabine in combination with other drugs [19–21] and a few bioanalytical methods are also reported [22]. However, until now, there have been no published reports nor data on the optimization using central composite design (CCD) of these chromatographic methods, and *in vitro* and *in vivo* release of emtricitabine NPs have been reported to investigate the content by HPLC.

The purpose of the present study was to develop and validate a simple and time-saving RP-HPLC method with UV detection for the determination of emtricitabine. The validated method was applied to quantify the content of emtricitabine incorporated into the PLGA nanoparticles after preparation. This is the first comprehensive study to investigate the content in *in vitro* and *in vivo* release of emtricitabine NPs by using optimized HPLC method. The method was validated according to Food and Drug Administration (FDA) and International Conference on Harmonization (ICH) guidelines [29, 30]. Lamivudine (0.2 *μ*g/mL) was used as an internal standard (IS).

## 2. Materials and Methods

Emtricitabine and lamivudine (Figure 1(b)) (99.8% w/w and 98.7% w/w, HPLC) were provided ex-gratis by M/S Cipla Laboratories, Mumbai, India. HPLC grade methanol was purchased from SD Fine-Chem Limited (Mumbai, India). Poly(lactic-co-glycolic acid) in a 50 : 50 molar ratio (M.W 14,500 Da) and an inherent viscosity of 0.53 dL/g (Resomer RG 504 H) was received as gift sample from M/s Boehringer Ingelheim Pharma GmbH & Co. KG (Binger Str. Ingelheim, Germany). Cetrimide was purchased from M/s SD Fine-Chem Limited (Mumbai, India). Dichloromethane was purchased from M/s Sigma-Aldrich (Mumbai, India). Deionized water used in all the experiments was passed through a Milli-Q water purification system (18.2 MΩ/cm), Millipore (Bangalore, Karnataka, India).

### 2.1. Instrumentation and Chromatographic Conditions

The HPLC (Shimadzu, Kyoto, Japan) instrument was equipped with two LC-10 ATVP pumps, SPD-10AVP UV-vis detector, Rheodyne injector with a 50 *μ*L loop. The column used for the analysis was a Phenomenex C_18_ (250 mm × 4.6 mm I.D., 5 *μ*m) column, supported with a Security Guard cartridge Phenomenex_ (Torrance, USA), with 3.0 mm internal diameter, in an oven at a temperature of 35°C. The results were acquired and processed using Shimadzu LC-solution version 6.42 software for data acquisition and processing. Chromatographic analysis was conducted in isocratic mode. The detection was carried out at 280 nm. An injection volume of 10 *μ*L was used for all standards and samples.

### 2.2. Experimental Design for HPLC Separation Optimization

Statistical parameter evaluation and experimental design are two major tools for optimization techniques. It is beneficial to evaluate and identify the most imperative parameters with a minimum number of runs, while using an appropriate model. The choice of the proper parameter levels through trial-and-error experiments is a time-consuming process, from which the optimal parameter settings may not readily be obtained.

There were three steps of the optimization of HPLC method:

- preliminary experiments to choose essential requirements of the method,
- screening to select important variables,
- response surfacing to locate the optimal point. During the optimization steps, retention time, peak resolution, and peak asymmetry responses were screened in order to minimize the analysis time and maximize the peak resolution and optimal peak asymmetry of the developed method.

Taguchi orthogonal array design was employed in preliminary experiments to screen the most appropriate parameters. Furthermore, we selected a CCD to determine the best experimental conditions in RP-HPLC. Thirteen experiments were conducted using the levels described in Table 1 and conditions described in Table 2. Minimum and maximum values for concentration of methanol (*A*) were selected as 10% and 30%, respectively. Likewise, minimum and maximum contents of buffer pH (*B*) were fixed as 2.5 and 4.0, respectively. Retention times  (*Y*_1_), peak resolution (*Y*_2_), and peak asymmetry (*Y*_3_) were the responses for these studies.

### 2.3. Preparation of Calibration Curve (CC) and Quality Control Samples (QC)

Eight-point calibration curve (CC) was prepared by serial dilution of emtricitabine stock solution (100 *μ*g/mL) in the range of 0.040, 0.1, 0.2, 0.4, 0.8, 1.2, 1.6, and 2 *μ*g/mL obtained by measuring the required amount of 100 *μ*g/mL working standard solution, mixing with a sufficient quantity of mobile phase, and making up to 10 mL. Calibration standards were prepared daily by spiking 0.1 mL of blank plasma with 10 *μ*L of the appropriate working solution resulting in concentrations of 0.040, 0.1, 0.2, 0.4, 0.8, 1.2, 1.6, and 2 *μ*g/mL. Stock solution (0.2 *μ*g/mL) of lamivudine (I.S) in methanol was prepared and stored at −20°C. The stock and standard solutions were prepared on a daily basis and stored in the dark at about 5°C. All solutions were used on the day they were prepared.

For the determination of the limit of detection (LOD) and limit of quantitation (LOQ) of the method, six standard solutions, between 0.005 and 0.040 *μ*g/mL, were obtained from the 10 *μ*g/mL working solution. All stock solutions were stored at −20°C and working solutions were freshly prepared each day.

### 2.4. Sample Preparation

To a 100 *μ*L of rat plasma, 10 *μ*L of IS and 150 *μ*L of emtricitabine were added and the mixture was incubated at 37°C for 1 h. Emtricitabine was then extracted using 100 *μ*L of acetonitrile (liquid-liquid extraction; LLE) followed by vortexing for 2 min. After vortexing, the samples were subjected to centrifuge at 12,000 ×g for 15 min. Supernatant was decanted and dried, using nitrogen gas at 50°C. Dry sample was reconstituted in the mobile phase and transferred to the autosampler vials and subjected to HPLC for analysis.

### 2.5. Method Validation

The parameters considered for the validation included selectivity and specificity, linearity, accuracy, precision, recovery, limits of detection and quantitation, system suitability, and stability.

### 2.6. System Suitability Tests

The system suitability parameters were determined by injecting six times the standard solution containing emtricitabine at a concentration of 1.6 *μ*g/mL. Other chromatographic parameters, such as capacity factor (*k*′), resolution (*R*) tailing factor (*T*), and theoretical plate number (*N*), were also analyzed. The capacity factor is a measure of where the peak of interest is located with respect to the void volume, that is, corresponding to the elution time of the nonretained components.

*R* is a measure of the degree of separation of two peaks. The tailing factor is a measure of the peak symmetry, and the theoretical plate number is a measure of the column efficiency, that is, how many peaks can be located per unit run-time of the chromatogram. The capacity factor, injection repeatability, tailing factor, theoretical plate number, and resolution for the two antiretroviral drug peaks were the constraints tested on a combination solution containing 1.6 *μ*g/mL of emtricitabine and 0.2 *μ*g/mL of IS.

### 2.7. Limits of Detection and Quantification

The limits of detection and quantitation were determined based on a specific calibration curve obtained from six standard solutions (0.005, 0.010, 0.020, 0.030, 0.035, and 0.040 *μ*g/mL) at concentrations in the proximity of these limits values. LOD and LOQ were calculated according to LOD = 3.3 *σ*/S and LOQ = 10 *σ*/S, where *σ* is the standard deviation of the response and S is the slope of the calibration curve.

### 2.8. Linearity

Calibration curves were constructed with eight standard solutions, containing the three compounds simultaneously, ranging from 0.040 to 2.0 *μ*g/mL. Linearity was determined through the calculation of a regression line by the method of least squares, representing the peak area as a function of the standard concentration. Data collected were analyzed using the Analysis ToolPak of Microsoft Excel (Microsoft Corp., Redmond, WA) with linear regression by the least squares method. The analysis of the response factors, that is, the peak area divided by the concentration of each standard, was also considered.

### 2.9. Accuracy and Precision

Precision indicates the closeness of agreement, that is, the degree of scatter between a series of measurements obtained from multiple sampling of the same homogeneous sample and it was determined by repeatability (intraday) and intermediate precision (interday) for three consecutive days. Four standard solutions (quality controls), 0.1, 0.8, 1.6, and 2.0 *μ*g/mL, respectively, were prepared six times each and analyzed according to the proposed method (intraday precision) for three consecutive days (interday precision). The relative standard deviation (RSD) determined at each concentration level should not exceed 15%, except for the lower limit of quantitation, where it should not exceed 20% [31].

The accuracy of the method expresses the closeness of agreement between the true value and the value found. It was determined by measuring six replicates of the four quality controls and by calculating the percentage of bias for each compound according to the equation % accuracy = (observed concentration/nominal concentration) × 100. The mean value should be within 15% of the actual value, except at the LOQ, where it should not deviate by more than 20% [32].

### 2.10. Method Applicability

#### 2.10.1. Preparation of Emtricitabine Loaded PLGA NPs

Emtricitabine NPs were prepared by solvent evaporation method double (multiple) emulsion process employing Ultra Turrax IKA T25 digital high shear homogenizer. First, an appropriate amount of emtricitabine was dissolved in 30 mL of aqueous phase and then this drug solution was added to organic phase (50 mL) consisting of PLGA solution in dichloromethane with vigorous stirring to yield a water-in-oil emulsion. Next, the water-in-oil primary emulsion was added to 30 mL of (0.3%) cetrimide aqueous solution and further emulsified for around 30 min at appropriate stress mixing conditions to yield a water-in-oil-in-water (w/o/w) emulsion. The organic solvent was then allowed to evaporate at room temperature. The formed nanosuspension was centrifuged at 16,000 ×g for 1 hr, at 4°C. The supernatant was collected for dosing, and the pellet was resuspended in 20 mL of water in order to wash unencapsulated emtricitabine and cetrimide. Placebo NPs were prepared following the above method without inclusion of emtricitabine.

#### 2.10.2. Particle Size (*D*_*nm*_), Polydispersity Index (PDI), and Zeta (*ζ*) Potential Measurements

The *D*_*nm*_ and PDI of the *t*-RVT NPs were determined using Malvern Zetasizer Nano S90 (Malvern Instruments Ltd., Worcestershire, U.K) and the zeta potential was measured using Malvern Zetasizer Nano ZS (Malvern Instruments Ltd., Worcestershire, UK). Samples were diluted in Milli-Q water before measurement.

#### 2.10.3. Determination of Encapsulation Efficiency and Drug Loading

The encapsulation efficiency of NPs was determined by the separation of drug-loaded NPs from the aqueous medium containing nonassociated emtricitabine by centrifugation (REMI high speed, REMI Corporation, India) at 12,000 ×g for 30 min, at 4°C. The amount of emtricitabine loaded into the NPs was calculated as the difference between the total amount used to prepare the NPs and the amount that was found in the supernatant. The amount of free emtricitabine in the supernatant was determined in triplicate by HPLC. The encapsulation efficiency of the NPs was determined in triplicate and calculated as follows:

(2) %  encapsulation  efficiency   =((Weight  of  drug  added  during  NP  preparation   −Weight  of  free  drug  in  supernatant)×100)  ×(Weight  of  drug  added  during  NP  preparation)−1.

For estimation of drug loading, equivalent 10 mg of NP containing emtricitabine was dissolved in 10 mL of methanol and analyzed by HPLC. The drug loading was determined using the following formula:

(3) $$\begin{array}{c} \%\;\text{Drug}\;\text{loading}=\frac{\text{Weight}\;\text{of}\;\text{drug}\;\text{in}\;\text{NPs}}{\text{Weight}\;\text{of}\;\text{NPs}\;\text{recovered}}\times 100. \end{array}$$

### 2.11. *In Vitro* Drug Release Studies

Dialysis membrane method was used to determine the release of emtricitabine from the NPs formulation. Freshly made emtricitabine loaded PLGA NPs were separated from the aqueous NPs suspension medium through ultra-centrifugation. These NPs were dried at room temperature for 12 h. NPs equivalent to one dose of emtricitabine (200 mg) were then redispersed in 1 mL of purified water and placed in a dialysis bag (molecular weight cut-off 10,000–12,000 Da, Hi-Media, India), which was tied and placed into 200 mL of dissolution media. The entire system was kept at 37 ± 0.5°C with continuous magnetic stirring (25 rpm) and the study was carried out in an adequate dissolution medium in phosphate buffered saline (PBS) (pH 7.4) for 15 days. At appropriate time intervals, aliquots of 1 mL were collected and filtered by 0.22 *μ*m membranes to remove NPs in suspension and replaced with 1 mL of fresh buffer. The amount of emtricitabine in the resulting samples was determined by the described HPLC method at 280 nm. The studies were performed in triplicate.

### 2.12. *In Vivo* Pharmacokinetic Studies in Rat

The pharmacokinetic studies were carried out in healthy male Wistar rats (250–300 g). The animals were fasted overnight before dosing with free access to water. The animals were acclimatized to laboratory conditions over the week before experiments and fed with standard rat diet, under controlled conditions of a 12 : 12 h light : dark cycle, with a temperature of 22 ± 3°C and relative humidity of 50 ± 5% RH. The experimental protocol was approved by the Institutional Animal Ethical Committee (AACP/IAEC/Jun-2012-02).

Eighteen rats were randomly separated into three groups (six animals in each group). The grouping of animals was as follows:

Group  I: control normal rats (received saline solution)
Group  II: administered with pure drug (as solution) (15 mg/kg/rat) [33],
Group  III: administered with emtricitabine NPs (as dispersion in 1 mL of water) and then administered orally using oral gavage needle (No18).

At regular time intervals 0, 1, 2, 4, 6, 8, 12, and every 24 h, for 10 days, samples of blood were withdrawn (100 *μ*L) from the retroorbital plexus by microcapillary technique under light ether anesthesia into heparinized microcentrifuge tubes (50 units heparin/mL of blood). Plasma was separated by centrifugation at 12,000 ×g for 15 min and analyzed by the following method.

Plasma samples were deproteinated with 1 mL of acetonitrile, vortexed for 30 s, and centrifuged at 8,000 ×g for 15 min. The supernatant was decanted into a China dish and evaporated to dryness at room temperature. This was further reconstituted with 100 *μ*L of mobile phase and vortexed for 30 s and 20 *μ*L was injected into an HPLC system. Emtricitabine was detected at a wavelength of 280 nm. The proficiency of nanoparticulate formulations was appraised by administering pure drug orally and measuring the blood levels at 0, 0.25, 0.5, 1.5, 2, 3, 4, 6, 8, 12 and 24 h.

### 2.13. Statistical Analysis

Data were given as the mean ± SEM Emtricitabine concentrations were expressed in *μ*g/mL. A commercially available package (Prism version 5.00; GraphPad Software Inc., San Diego, CA) was used for all statistics. Data was evaluated by two-tailed Unpaired *t*-test with Welch's correction (GraphPad Prism). A *P* < 0.01 level was taken as significant.

## 3. Results and Discussion

### 3.1. Optimization of Separation

The development of an optimized method requires plenty of experiments that increase exponentially with the number of independent variables. To decrease the number of experiments, a decrease in dimensions of independent variables was considered in a series of preliminary-screening experiments. Optimization of the chromatographic method was achieved in three steps: a series of preliminary experiments followed by two sets of different experiments. The experimental designs were performed to achieve maximum resolution in short analysis time and optimal peak asymmetry.

### 3.2. Preliminary Studies

The preliminary experiments were executed to decide the essential analytical requirements of the method, such as the type of column, buffer, and pH range. A standard solution containing 2 *μ*g/mL of emtricitabine was used during the initial experiments. In our preliminary study, performance of several kinds of columns (Hypersil C_18_ (200 mm × 4.6 mm, 5 *μ*m), Grace smart C_18_ (150 mm × 4.6 mm, 5 *μ*m), Waters Symmetry C_18_ (200 mm × 4.6 mm, 5 *μ*m), and Phenomenex C_18_ (250 mm × 4.6 mm, 5 *μ*m)) was checked by running dissimilar mobile phases. The best peak asymmetry and peak resolution were obtained with Phenomenex C_18_. Therefore, Phenomenex C_18_ was selected as the analytical column. The resolution of Phenomenex C_18_ was higher and showed much better peak asymmetry than with other columns.

Three different buffers, ammonium acetate, potassium dihydrogen phosphate, and sodium dihydrogen phosphate, were used and it was found that potassium dihydrogen phosphate improved the peak shape of emtricitabine and produced the best resolution.

### 3.3. Screening Based on a Taguchi Orthogonal Array Design

If the number of factors is high, an absolute response surface would be a complex multidimensional structure needing much more experimental research in order to be fully determined. Thus a screening study was applied to choose momentous parameters on separation. Taguchi orthogonal array design permitted evaluation of whether variables have a considerable influence on the chosen response or not. The parameters considered in the Taguchi orthogonal array design were the methanol percentage, mobile phase ratio and pH, injection volume, and flow rate. The experimental ranges of the variables were elected on the basis of preliminary experiments. The distincted responses of the variables were the retention times, peak resolution, and peak asymmetry.

**Figure 2:**
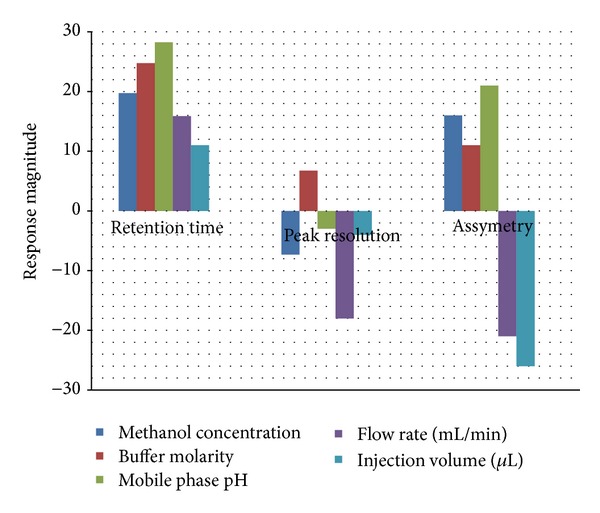
Influence of selected parameters on the response magnitude.

As can be seen in Figure 2, methanol concentration, buffer molarity, flow rate, and injection volume had a negative effect on peak resolution and peak asymmetry while all the above parameters had a positive effect on retention time.

Methanol concentration and mobile phase pH had significant effect on retention time and peak asymmetry and were selected for further optimization. Buffer molarity was not selected for examination because of its positive effect on all of the three variables. It was, therefore, fixed to its maximum value (40 mM). The flow rate and injection volume were fixed to their optimum levels, which were 1 mL/min and 20 *μ*L, respectively, to decrease analysis time and maximize resolution.

### 3.4. Response Surfacing Based on 3^2^ Central Composite Design

Response surface mapping was an efficient way to find the optimum condition. In this case, the 2-factor-3-level CCD was employed to draw response surface graphs to determine the optimal conditions and to investigate parabolic interactions between parameters (methanol concentration and mobile phase pH). The variables with their relative experimental values are reported in Table 1. This design permitted the response surface to be modeled by fitting a second-order polynomial with the number of experiments equal to 2^*k*^ + 2*k* + 1, where *k* is the number of variables, which composed a total of 13 experiments to be executed as per CCD design (Table 2). Experiments were executed according to the design listed in Table 2 and responses measured are given in the same table.

**Figure 3:**
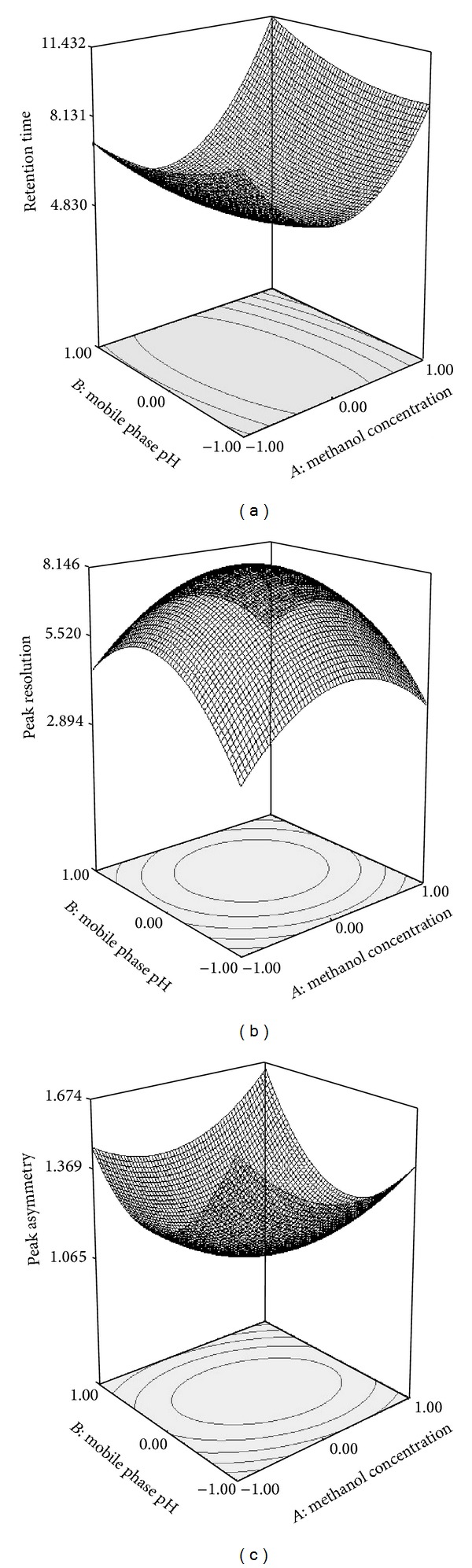
Three-dimensional graph showing (a) the effect of mobile phase and methanol concentration on retention time. (b) Three-dimensional graph showing the effect of mobile phase and methanol concentration on peak resolution. (c) Three-dimensional graph showing the effect of mobile phase and methanol concentration on peak asymmetry.

Three-dimensional surface plots are presented in Figure 3 and are extremely valuable for studying the interaction effects of the factors on the responses. The retention time for emtricitabine decreases as the methanol (*v*/*v*)%, augmented from lower to intermediate level (Figure 3(a)), when the buffer molarity was kept at constant 40 mM and pH of mobile phase was at intermediate level. An augment in buffer molarity at constant pH and constant methanol (*v*/*v*)% results in decrease in the retention time of emtricitabine, most likely due to the escalating competition of buffer cations for silanol sites which are preferentially attached to the column. This effect is important when the buffer molarity is greater than 40 mM. The effect of mobile phase pH on the retention time of emtricitabine was therefore investigated in a pH range from 2.7 to 3.9. Retention time was considered a more critical parameter in terms of analytical run time and sampling throughout analysis.

A classical second-degree model with a 3D experimental domain was hypothesized. The coefficients for the second-order polynomial model were estimated by least squares regression. The equation for the *Y*_1_ (retention time) factor is shown in (4). The regression coefficients calculated from CCD are given as follows:

(4) Y1=4.931+1.203x1+0.231x2+3.538x1x2+0.6739x12+0.852x22.

The optimized chromatographic conditions were then used for all future analytical studies.

To establish peak asymmetry, a line was drawn through the peaks generated following analysis of samples. In general, peak symmetry was improved at intermediate level of mobile phase pH and methanol concentration as shown in Figure 3(b).

Peak resolution was extensively affected when the mobile phase pH and methanol concentration decreased to low level as depicted in Figure 3(c). This result designates that both methanol concentration and mobile phase pH were one of the most important parameters that can be manipulated to optimize the separation and analysis of emtricitabine. In the mobile phase pH range investigated, the resolution of emtricitabine was improved as the pH was increased from lower to intermediate level. The effect of mobile phase pH and methanol concentration on peak resolution is depicted in Figure 3(c). When using a methanol concentration, mobile phase pH at intermediate level resulted in improved peak resolution. As shown in Figure 3(c), a decrease in resolution was observed as mobile phase of lower pH was used. When the methanol concentration was at intermediate level peak resolution was improved.

The mathematical relationship in the form of polynomial equations for the measured responses *Y*_2_ and *Y*_3_ is given as follows:

(5) Y2=8.082+0.268x1+0.700x2−1.882x1x2−2.177x12−0.160x22,Y3=1.065−0.016x1+0.00x2+0.170x1x2+0.320x12+0.100x22.

The model was authenticated by analysis of variance (ANOVA) employing Design Expert software that had been used to develop the experimental plan for RSM. The ANOVA tests demonstrated that the models materialized to be adequate, with significant lack of fit (*P* < 0.0001) and with a satisfactory coefficient of correlation (*r*).

It should be noted that the peak asymmetry achieved with the optimized chromatographic conditions was 1.1 and was considered suitable for this method. The final optimum conditions for chromatographic separation were 40 mM buffer molarity, pH 3.2, and methanol concentration 15%. The optimized mobile phase consisted of a mixture of 40 mM phosphate dihydrogen phosphate buffer (pH 6.8), methanol, and 2% acetonitrile in a ratio of 83 : 15 : 2, v/v/v; at a flow rate of 1 mL/min.

### 3.5. Method Validation

#### 3.5.1. System Suitability Tests

To assure the feasibility and adequacy of the proposed method for estimation of emtricitabine in routine pharmaceutical application and verify the resolution, column efficiency, and chromatographic repeatability, system suitability tests were performed (Table 3). The capacity factor (*k*′) was between 1 and 10, indicating good resolution with respect to the void volume. The RSD of peak areas of six consecutive injections was found to be less than 2%, thus showing good injection repeatability and excellent chromatographic and environmental conditions. The tailing factor (*T*) for the emtricitabine was found to be close to 1, reflecting good peak asymmetry. The resolution (*R*_*s*_) between the peaks was found to be greater than 2, indicating good separation of the emtricitabine. The values for theoretical plate number (*N*) demonstrated good column efficiency. Resolution between emtricitabine and lamivudine was 7.32.

#### 3.5.2. The Limit of Detection (LOD) and Quantitation (LOQ)

The lowest concentration at which an analyte can be detected or quantified with acceptable precision and accuracy can be determined by different methodologies. The estimated LOD and LOQ for emtricitabine were 0.024 *μ*g/mL and 0.036 *μ*g/mL, respectively.

#### 3.5.3. Linearity

Linearity was evaluated over the concentration range 0.040–2.0 *μ*g/mL for emtricitabine, estimating the regression equation and the determination coefficients (*R* ^2^) obtained from the least squares method. The coefficients of determination for the calibration curves of the three compounds were higher than 0.9996, which is generally considered as evidence of an acceptable fit of the data to the regression line and indicating good linearity over the concentration range proposed.

#### 3.5.4. Precision and Accuracy

Accuracy and precision for the quality controls in the intraday and interday run are shown in Table 4. All of the data fulfill the acceptance criteria. The intra- and interday RSD values did not exceed 5.0%. The intra- and interday bias values were found in the interval 3.0 to −5.0%. These data indicate that the developed method is accurate, reliable, and reproducible, since neither RSD nor bias exceeded 15%, which is in agreement with acceptance recommendations.

#### 3.5.5. Specificity

**Figure 4:**
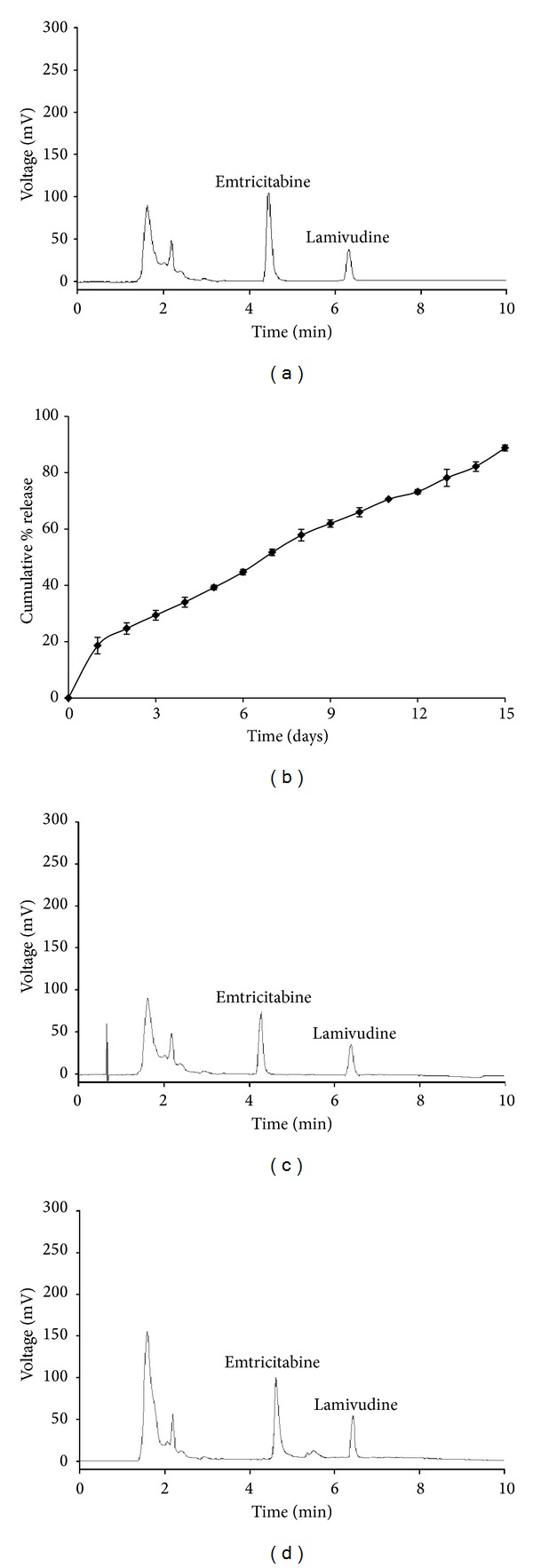
Optimized conditions: (a) chromatographic profile of the plasma spiked with emtricitabine (2 *μ*g/mL) in the presence of the I.S lamivudine 0.2 *μ*g/mL at pH value of 3.2 and identical compositions of mobile phase (phosphate dihydrogen phosphate buffer : methanol (40 mM) (85 : 15 v/v)) at a flow rate of 1 mL/min. (b) *In vitro* release profiles of emtricitabine loaded PLGA (50 : 50) (M.W 14,500 da) nanoparticles with cetrimide as stabilizer in pH 7.4 phosphate buffer. Data points shown are mean ± standard deviation (*n* = 3). (c) Chromatogram of plasma sample collected from rats 2 h after administration of free emtricitabine. (d) Chromatogram of plasma sample collected from rats 16 h after receiving oral administration of emtricitabine NPs.

Specificity is expressed as the capability of a method to distinguish the analyte from all potentially intrusive substances. The specificity of the method was scrutinized by blank plasma detection, peak purity, and spiking blank plasma with pure standard compounds. Blank rat plasma had no interference, when emtricitabine and the IS were eluted. At optimized conditions, the separation of emtricitabine and lamivudine was completed within 7 min (Figure 4(a)).

#### 3.5.6. Stability

Bench-top stability was investigated to ensure that emtricitabine was not degraded in plasma samples at room temperature for a time period to cover the sample preparation. It was measured by divulging the QC samples to ambient laboratory conditions for 10 h. Freeze-thaw stability was measured over three cycles. Because of the need for occasional delayed injection of extraction samples, the stability of reconstituted samples was assessed at ambient temperature for 24 h. The freezer storage stability of emtricitabine in rat plasma at −20°C was evaluated by assaying QC samples at the beginning and one week later. All stability QC samples were analyzed in six replicates. The results indicated that emtricitabine had an acceptable stability under those conditions (Table 5).

### 3.6. Method Applicability

The method developed in this work was used to determine the content of emtricitabine in *in vitro* drug and *in vivo* release in free form and as NPs.

Developed emtricitabine NPs had a Z-average 180 nm, polydispersity index (PDI) of 0.073, and zeta potential of −29 mV. Percentage encapsulation efficiency (EE) and percentage drug loading for emtricitabine were found to be 74.34% and 95.44%, respectively (*n* = 3), representing a high degree of EE of emtricitabine into PLGA NPs. Emtricitabine has high solubility in water and it is necessary to prepare the NPs employing w/o/w emulsion technique, in order to augment the EE of this molecule. The *in vitro* drug release profile showed a 15% initial burst in the first day, followed by 80% accumulative drug release of emtricitabine after 15 days (Figure 4(b)) in the PBS buffer at pH 7.4. Zero-order patterns were observed of optimized NPs formulation with *R* ^2^ values of 0.9904.

**Figure 5:**
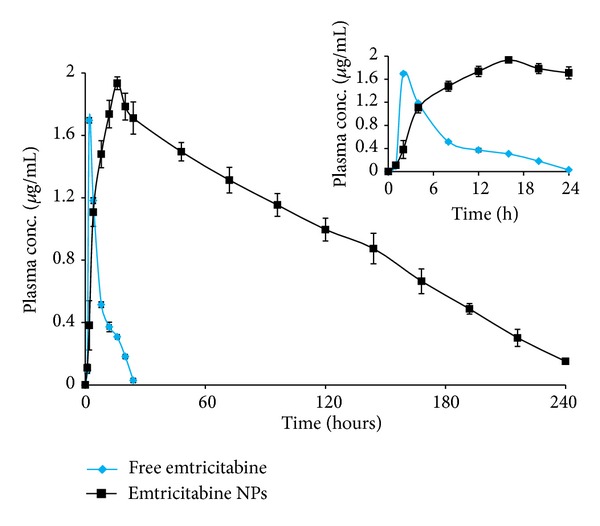
Plasma concentration-time curve of free emtricitabine and emtricitabine NPs after being orally administered in male Wistar rats (*n* = 6, mean ± S.D). The inset shows the Plasma concentration-time curve in 24 h.

The areas under the concentration versus time curves were 14.83 *μ*g/mL∗h and 238.66 *μ*g/mL∗h for free emtricitabine and emtricitabine NPs, respectively. Oral administration of emtricitabine in the present study resulted in a sharp *C*_max⁡_ of 1.696 *μ*g/mL within 2 h after which the plasma concentration declined rapidly, indicating a rapid absorption of emtricitabine, whereas a relatively slow increase and sustained plasma concentration of emtricitabine was observed for a longer time (10 days) after the administration of a single dose of emtricitabine NPs. Significantly (*P* < 0.05) high *C*_max⁡_ of 1.934 *μ*g/mL at 16 h with emtricitabine still detectable after 10 days confirms the sustained effect of polymeric NPs. The representative chromatogram of a plasma sample, which was collected from a Wistar rats at 2 h following oral administration of free emtricitabine (Figure 4(c)) and at 16 h of emtricitabine NPs (Figure 4(d)). The mean plasma concentration-time profiles after an oral administration of free emtricitabine and emtricitabine NPs are shown in Figure 5.

The pharmacokinetic data of free emtricitabine and emtricitabine NPs after oral administration in rats is shown in Table 6.

### 3.7. Statistical Analysis

**Figure 6:**
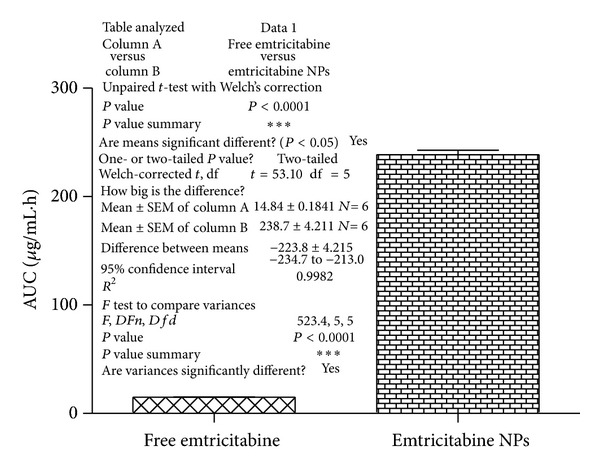
Comparison and effect of free emtricitabine and emtricitabine NPs on AUC_0−*∞*_. Statistics: comparison based on AUC_0−*∞*_ by two-tailed unpaired *t*-test with Welch's correlation test ***P < 0.001.

Two-tailed unpaired *t*-test with Welch's correction was performed by using GraphPad Prism. The AUC values obtained from free drug were compared with those obtained from emtricitabine NPs in Wistar rats. Significant difference (***P < 0.01) was found in the *in vivo *profile of emtricitabine NPs when compared with free emtricitabine (Figure 6). The concentration values of drug versus time, for free emtricitabine and emtricitabine NPs, were subjected to two-tailed unpaired *t*-test and the *P*  values (***P < 0.001) indicated extremely significant difference.

## 4. Conclusions

The novelty of the current work is the development of nanoparticle drug delivery system solely through the judicious selection of apt blend of PLGA and emulsifier and evaluate its* in vitro* release and *in vivo *absorption performance by systematically optimized HPLC method using Formulation by Design (FbD). Experimental designs have been employed during the development of the method to minimize retention time and maximize peak resolution and optimal peak asymmetry. The predicted values from the model equation were found to be in good agreement with observed values and to gain a better understanding of the two variables. Finally, the method was applied to investigate the content of emtricitabine in *in vitro *drug and *in vivo* release studies in free form and as NPs. Considerable enhancement in the rate and extent of oral drug absorption ratified the superior performance of the nanoparticle drug delivery system in enhancing the bioavailability of emtricitabine. Conclusively, the studies can be judiciously explored to develop suitable platform technology (ies) for development of effectual and cost-effectual optimized HPLC method to investigate the * in vitro * and *in vivo *release performance of nanoparticle drug delivery system of other drugs.

## Figures and Tables

**Table 1:** Independent variables, dependent variables, and levels of the face centered central composite design.

Factor	Symbol	Level (−1)	Level (0)	Level (+1)
Independent				
Methanol concentration (%)	*A*	10	20	30
Mobile phase pH	*B*	2.5	3.5	4.0
Dependent				
Retention time	*Y*_1_			
Peak resolution	*Y*_2_			
Peak asymmetry	*Y*_3_			

**Table 2:** Experimental conditions according to the central composite design and observed response values.

Exp. no.	Run order	*A*	*B*	*Y*_1_	*Y*_2_	*Y*_3_
1	8	−1.00	−1.00	9.21	2.52	1.6
2	3	1.00	−1.00	7.64	4.74	1.4
3	4	−1.00	1.00	8.57	3.11	1.6
4	11	1.00	1.00	10.41	4.69	1.8
5	10	−1.00	0.00	5.37	7.81	1.2
6	12	1.00	0.00	12.32	5.62	1.1
7	9	0.00	−1.00	6.35	4.59	1.5
8	7	0.00	1.00	5.61	8.25	1.1
9	2	0.00	0.00	4.76	7.64	1.1
10	1	0.00	0.00	4.79	8.01	1.2
11	5	0.00	0.00	4.77	7.78	1.0
12	6	0.00	0.00	4.81	8.11	1.1
13	13	0.00	0.00	4.78	7.85	1.1

*A*: methanol concentration (%); *B*: mobile phase pH.

*Y*_1_: retention times; *Y*_2_: peak resolution; *Y*_3_: peak asymmetry.

**Table 3:** System suitability parameters.

Parameter	Compound	
	Emtricitabine	IS
Retention time (Rt)	4.39	6.58
Tailing factor (*T*)	1.01	1.13
^†^Injection repeatability (RSD)	0.752	0.623
^‡^Resolution (*R*_*s*_)	—	7.32
Capacity factor (*K*′)	5.92	6.30
Theoretical plates (*N*)	4976	5318
Asymmetry	1.23	1.36

^†^RSD of peak areas of six consecutive injections at a concentration of 2.0 and 0.2 µg/mL of emtricitabine and IS, respectively.

^‡^Resolution between emtricitabine and IS.

**Table 4:** Intraday and interday precision and accuracy of emtricitabine in rat plasma (*n* = 6).

Concentration (µg/mL)	Observed concentration (µg/mL)	% precision	% accuracy
Intraday			
0.1	0.094 ± 0.002	2.12	94
0.8	0.781 ± 0.031	3.96	97.62
1.6	1.592 ± 0.056	3.51	99.50
2.0	1.985 ± 0.110	5.54	99.25
Interday			
0.1	0.089 ± 0.007	7.86	89
0.8	0.725 ± 0.038	5.24	90.62
1.6	1.512 ± 0.089	5.88	94.50
2.0	1.920 ± 0.135	7.03	96

**Table 5:** Stability of emtricitabinein rat plasma (*n* = 6).

Sample condition	Spiked concentration (*μ*g/mL)	Mean determined concentration (µg/mL)	Accuracy (%)
Bench-top stability^♦^	0.1	0.096	96.0
	0.8	0.783	97.87
	1.6	1.571	98.18
	2.0	1.944	97.20

Freeze-thaw stability^*♣*^	0.1	0.099	99.00
	0.8	0.795	99.37
	1.6	1.582	98.87
	2.0	1.995	99.75

One-week stability^▲^	0.1	0.093	93.00
	0.8	0.746	93.25
	1.6	1.517	94.81
	2.0	1.911	95.55

^*♦*^Exposed at ambient temperature (25°C) for 4 h.

^*♣*^After three freeze-thaw cycles.

^▲^Stored at −16°C.

**Table 6:** Pharmacokinetic parameters of free emtricitabine and emtricitabine NPs at a dose of 15 mg/kg/rat.

Pharmacokinetic parameters	Free emtricitabine	Emtricitabine NPs
*C*_max⁡_ (µg/mL)	1.696 ± 0.017	1.934 ± 0.041
*t*_max⁡_ (h)	2.0 ± 0.006	16 ± 0.031
*t*_1/2_ (h)	9.366 ± 0.580	32.099 ± 2.194
AUC_0–∞_ (µg/mL∗h)	14.835 ± 0.450	238.667 ± 10.315
AUMC_0–∞_ (µg/mL∗h)	178.32 ± 15.095	22156.689 ± 1243.02
*K*_*e*_ (1/h)	0.0741 ± 0.004	0.0215 ± 0.0013
MRT	12.0 ± 0.670	94.307 ± 1.950

Data presented as mean ± standard deviation (*n* = 6).
